# TB testing in HIV-positive patients prior to antiretroviral treatment

**DOI:** 10.5588/ijtld.21.0195

**Published:** 2022-03-01

**Authors:** E. Spooner, S. Reddy, S. Ntoyanto, Y. Sakadavan, T. Reddy, S. Mahomed, K. Mlisana, M. Dlamini, B. Daniels, N. Luthuli, N. Ngomane, P. Kiepiela, A. Coutsoudis

**Affiliations:** 1 Department of Paediatrics and Child Health, University of KwaZulu-Natal, Durban, South Africa; 2 HIV Prevention Research Unit, South African Medical Research Council, Durban, South Africa; 3 South African Medical Research Council, Durban, South Africa; 4 Biostatistics Unit, South African Medical Research Council, Durban, South Africa; 5 School of Laboratory Medicine and Medical Sciences, University of KwaZulu-Natal, Durban, South Africa; 6 Medical Microbiology Department, National Health Laboratory Services, Durban, South Africa; 7 Centre for AIDS Programme Research in South Africa, Durban, South Africa; 8 EThekwini Health Unit, EThekwini Municipality, Durban, South Africa; 9 Occupational Health, Durban, South Africa

**Keywords:** POCT, TB-LAMP, TB-LAM, Xpert® MTB/RIF, ART-naïve

## Abstract

**BACKGROUND:**

TB diagnosis in patients with HIV is challenging due to the lower sensitivities across tests. Molecular tests are preferred and the Xpert^®^ MTB/RIF assay has limitations in lower-income settings. We evaluated the performance of loop-mediated isothermal amplification (LAMP) and the lipoarabinomannan (LAM) test in HIV-positive, ART-naïve clinic patients.

**METHODS:**

A total of 783 eligible patients were enrolled; three spot sputum samples of 646 patients were tested using TB-LAMP, Xpert, smear microscopy and culture, while 649 patients had TB-LAM testing. Sensitivity, specificity, and negative and positive predictive values were estimated with 95% confidence intervals.

**RESULTS:**

Sensitivities for smear microscopy, TB-LAMP and Xpert were respectively 50%, 63% and 74% compared to culture, with specificities of respectively 99.2%, 98.5% and 97.5%. An additional eight were positive on TB-LAM alone. Seventy TB patients (9%) were detected using standard-of-care testing, an additional 27 (3%) were detected using study testing. Treatment was initiated in 57/70 (81%) clinic patients, but only in 56% (57/97) of all those with positive TB tests; 4/8 multidrug-resistant samples were detected using Xpert.

**CONCLUSION:**

TB diagnostics continue to miss cases in this high-burden setting. TB-LAMP was more sensitive than smear microscopy, and if followed by culture and drug susceptibility testing as required, can diagnose TB in HIV-positive patients. TB-LAM is a useful add-in test and both tests at the point-of-care would maximise yield.

## Abstract (fr)

**CONTEXTE:**

Le diagnostic de la TB chez les patients infectés par le VIH est difficile en raison de la faible sensibilité des tests. Les tests moléculaires sont préférés puisque l’utilisation du test Xpert^®^ MTB/RIF a des limites dans les pays à faible revenu. Nous avons évalué la performance de l’amplification isotherme induite par boucle (LAMP) et du test de détection du lipoarabinomannane (LAM) chez des patients cliniques positifs au VIH et naïfs de traitement antirétroviral.

**MÉTHODES:**

Au total, 783 patients éligibles ont été sélectionnés ; trois échantillons d’expectorations sur place de 646 patients ont été testés par test TB-LAMP, test Xpert, microscopie des frottis et culture, alors que 649 patients ont été testés par test TB-LAM. La sensibilité, la spécificité et les valeurs prédictives négatives et positives ont été estimées avec les intervalles de confiance à 95%.

**RÉSULTATS:**

La sensibilité de la microscopie des frottis, du test TB-LAMP et du test Xpert était respectivement de 50%, 63% et 74% par rapport à la culture, avec des spécificités respectives de 99,2%, 98,5% et 97,5%. Huit patients supplémentaires étaient positifs au seul test TB-LAM. Soixante-dix patients tuberculeux (9%) ont été détectés en utilisant le test de référence et 27 autres (3%) en utilisant les tests à l’étude. Un traitement a été instauré chez 57/70 (81%) patients cliniques, mais chez seulement 56% (57/97) de ceux dont les tests de dépistage de la TB étaient positifs ; 4/8 échantillons de TB multirésistante ont été détectés par test Xpert.

**CONCLUSION:**

Dans ce pays à forte incidence de TB, des cas de TB restent non-diagnostiqués par les tests diagnostiques. Le test TB-LAMP était plus sensible que la microscopie des frottis. S’il est suivi d’une culture et d’un test de sensibilité aux antituberculeux tel que requis, ce test peut permettre de diagnostiquer la TB chez les patients positifs au VIH. Le test TB-LAM est un test supplémentaire utile, et l’utilisation de ces deux tests au points d’intervention en maximiserait la portée.

There were 10 million TB cases and 1.4 million deaths worldwide in 2019;^1^ in South Africa, TB continued to be the leading cause of death in 2017.^2^ HIV and TB are hyper-endemic in South Africa, with nearly two-thirds of TB infections occurring in people living with HIV (PLHIV).^1^ KwaZulu-Natal (KZN) Province is the epicenter of both diseases, with the city of Durban in EThekwini registering the most cases.^3^

TB infection involves a dynamic interaction between host and pathogen that covers a spectrum of disease from latent to active infection; a median of 50% of subclinical infections is reported from prevalence surveys in Africa and Asia.^4^ TB is thus often undiagnosed and spreads undetected.^5^,^6^ HIV increases the risk of both reactivation and reinfection of TB disease by an estimated 18-fold; the risk of extrapulmonary disease is increased with advanced HIV disease.^7^

Rapid point-of-care testing (POCT) is the preferred TB diagnostic method, enabling prompt treatment initiation on the day of diagnosis to reduce infectivity, morbidity and loss to follow-up.^8^

Nucleic-acid amplification tests (NAAT) have improved TB diagnosis, with increased sensitivity compared to smear microscopy, and decreased testing turnaround times. Although the GeneXpert platform (Cepheid, Sunnyvale, CA, USA) was developed for POCT use in clinics, it has largely been placed in central laboratories throughout South Africa, removing the point-of-care benefit of the platform. The Xpert^®^ MTB/RIF test takes under 2 h, requires uninterrupted power supply, a computer and a temperature-controlled environment; also, the specimen cannot be used for further testing. In contrast, the Loopamp^TM^ MTBC Detection assay (Eiken Chemical Company, Tokyo, Japan) based on loop-mediated isothermal amplification (TB-LAMP) is a simple, more manual NAAT platform with visual fluorescent readout after 60 min that was developed for POCT TB diagnosis in peripheral resource-constrained settings. It requires less infrastructure and the same specimen can be used for smear, culture or line-probe assays (LPA).

Lipoarabinomannan (LAM) is a mycobacterial cell wall antigen released from active or degenerating bacteria, detected by a simple commercial urine lateral flow antigen POCT (Alere Determinee TB-LAM Ag; Abbott Laboratories, Abbott Park, IL, USA) processed at room temperature in 25 min. It is most sensitive in those with advanced HIV disease (CD4 <200 cells/mm^3^), who are often sputum-scarce; urine is thus a convenient, easier specimen to collect. The WHO has updated its recommendations for its use in HIV-positive patients.^9^ TB-LAM has demonstrated excellent specificity, and increases the yield of positive patients due to the added advantage of detecting extrapulmonary TB.^10^,^11^

TB-LAMP has been evaluated in patients suspected of having TB in Durban, South Africa.^12^ After this evaluation, a follow-on evaluation was undertaken in antiretroviral treatment (ART) naïve PLHIV at three primary healthcare (PHC) clinics to assess the performance of TB-LAMP sputum and TB-LAM urine testing in this high-burden population.

## METHODS

### Study design and population

A prospective descriptive study was undertaken from May 2014 to April 2015. ART-naive, PLHIV presenting for TB testing were recruited from Lancers Road Clinic (*n* = 518) and from Prince Cyril Zulu Communicable Diseases Centre (CDC) (*n* = 41), both in the centre of Durban’s major taxi rank. Additionally, 255 patients were recruited from Chesterville Clinic, a peri-urban community, 8 km from the city centre.

### Study and laboratory procedures

**Figure 1:**
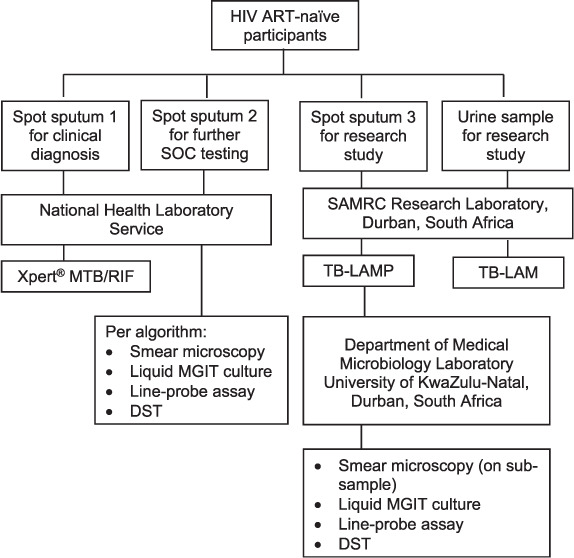
Flowchart of TB sample collection and tests performed at different laboratories. Samples were all collected consecutively on the same day. ART = antiretroviral therapy; SOC = standard of care; SAMRC = South African Medical Research Council; LAMP = loop-mediated isothermal amplification; LAM = lipoarabinomannan; MGIT = Mycobacterial Growth Indicator Tube; DST = drug susceptibility testing.

Symptom screening was not routinely documented in any of the clinics and TB testing was done per healthcare worker (HCW) request. All HIV-positive, non-ART patients presenting for TB testing were eligible for study participation. KZN Department of health (DoH) TB screening tool/risk questionnaire for PLHIV and a sociodemographic study questionnaire were completed. Samples were collected per standard operating procedures and infection control guidelines. Sputum induction with hypertonic saline was offered to participants struggling to produce sputum. One or two spot sputum samples were taken for TB screening (Samples 1 and 2) using standard-of-care (SOC) testing. An additional spot sputum sample (Sample 3) and a urine sample were collected on the same day for study purposes. Samples were processed as shown in Figure 1.

SOC samples were sent to the National Health Laboratory Service (NHLS) laboratories for routine testing. Study sputum samples were processed at the South African Medical Research Council (SAMRC) laboratory using the TB-LAMP assay, as described previously.^13^ Remaining samples were sent to the Department of Medical Microbiology in the NHLS Laboratory, where they were cultured using liquid MGIT^TM^ (Mycobacterial Growth Indicator Tube; BD, Sparks, MD, USA) and incubated in the BACTEC^TM^ MGIT^TM^ 960 system. Urine samples were tested at the SAMRC laboratory using the Alere Determine TB-LAM Ag (Alere Inc) following the manufacturer’s instructions.

### Ethics

The study protocol was approved by the SAMRC Ethics Committee, Cape Town, South Africa (EC16-7/2013), the EThekwini Research Ethics Committee, Durban (M.1/1/2 23 September 2013) and the University of KwaZulu-Natal Biomedical Research Ethics Committee, Durban (BFC 251/16). Written informed consent was provided by participants.

### Statistical analysis

The χ^2^ or Fishers exact test, where appropriate, was used to test the association between TB status and categorical variables of interest. Diagnostic accuracy was summarised using sensitivity, specificity, negative (NPV) and positive predictive values (PPV) with binomial confidence intervals. All analysis was performed using Stata v15.0 (Stata Corp, College Station, TX, USA).

## RESULTS

### Patient characteristics

Sociodemographic data for the patients are represented in Table 1; overall, 31% of patients and 38% of TB-positive patients were male. The median CD4 count was 262 cell/mm^3^ (34% had a CD4 count <200 cells/mm^3^) and the mean was lower in the TB-positive group at 211 cell/mm^3^ (*P* = 0.096). There were more TB cases in those with lower CD4 counts (18%), decreasing to 7% in those with CD4 counts >500 cells/mm^3^.

**Table 1:** Characteristics of all ART-naïve participants with any test positive for TB

	All patients (*n* = 783) *n* (%)	TB-positive (*n* = 97) *n* (%)	TB-negative (*n* = 686) *n* (%)	*P*-value
Age, years, median [IQR] (*n* = 780)^*^	31 [26–38]	33 [27–40]	31 [26–37]	
Sex				
Female	540 (69)	60 (62)	480 (70)	
Male	243 (31)	37 (38)	206 (30)	
CD4, cells/mm^3^, median [IQR] (*n* = 753)^*^	262 [158–345]	211 [108–301]	268 [167–349]	
<100	114(15)	20/114 (18)	94/114 (82)	0.096
101–200	146 (19)	23/146 (16)	123/146 (84)	
201–350	316 (42)	34/316 (11)	282/316 (89)	
351–500	106 (14)	10/106 (9)	96/106 (91)	
>500	71 (9)	5/71 (7)	66/71 (93)	
Positive WHO symptom screen (*n* = 780)^*†^	640 (82)	90 (93)	550 (81)	0.003
Positive KZN DoH screen (*n* = 783)^‡^	685 (87)	92 (95)	593 (86)	0.019
History of previous TB	119 (15)	16 (16)	103 (15)	0.704
TB contact in the last year (*n* = 779)^*^	237 (30)	28 (29)	209 (30)	0.706
Alcohol consumption	175 (22)	20 (20)	155 (23)	0.662
Cigarette smoking (*n* = 782)^*^	125 (16)	17 (18)	108 (16)	0.658
How long has HIV status been known? (*n* = 782)^*^				
Recently 0–6 months	711 (91)	86 (90)	625 (91)	0.737
6 months–1 year	28 (4)	3 (3)	25 (4)	
1–3 years	23 (3)	5 (5)	18 (3)	
>3 years	20 (3)	2 (2)	18 (3)	

^*^ Data available.

^†^ WHO 4 screening questions, 2011.

^‡^ 8 questions in provincial health department questionnaire.

ART =antiretroviral therapy; IQR = interquartile range; KZN = KwaZulu-Natal; DoH = Department of Health.

### Symptom screening

The 2011 WHO 4 symptom screen had a sensitivity of 92.8% and a specificity of 19.5%. The KZN DoH screening tool had a sensitivity of 94.8% and a specificity of 13.6%. Among those with no symptoms, 7/97 (7%) of TB-positive patients would not have been tested if the WHO 4 symptom screen had been followed and 5/97 (5%) missed if the DoH screen had been followed. One patient with multidrug-resistant TB (MDR-TB), CD4 of 52 cells/mm^3^ and five different positive TB tests was asymptomatic.

### Previous TB

A history of previous TB was reported by 119/783 (15%; *P* = 0.704) participants with a median time of previous infection of 5 years (interquartile range [IQR] 2–11). Of these, 16/119 (13.5%) had a positive TB test in the study, that is, 16% (16/97) of all those with positive TB tests.

### Samples collected

**Figure 2:**
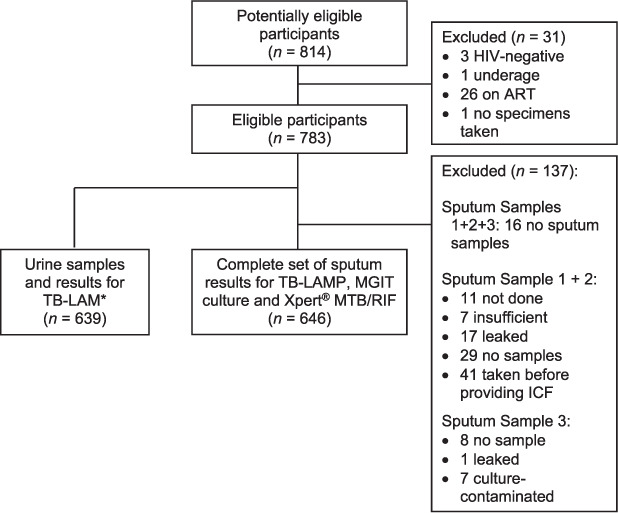
Flow diagram of enrolled HIV ART-naïve participants, losses, specimens and number of complete results. ^*^TB-LAM testing started 3 months into the study with amendment approval, after which all participants gave a urine sample. ART = antiretroviral therapy; LAM = lipoarabinomannan; LAMP = loop-mediated isothermal amplification; MGIT = Mycobacterial Growth Indicator Tube; ICF = informed consent form.

Of 783 participants, 56 (7%) required hypertonic saline nebulisation to assist with sputum production, 10 of whom could still not produce sputum. Figure 2 gives details on specimen collection.

### Test comparison

Table 2 shows the performance of the tests compared to A) culture as gold standard; B) Xpert as diagnostic standard; and C) NAAT test with smear-negative and smear-positive, culture-positive sputum. The median CD4 count in the 18/649 TB-LAM-positive patients was 79 cells/mm^3^ (IQR 36–263) compared to a median of 211 cells/mm^3^ (IQR 108–301) in other patients with positive results. Eight patients were positive on TB-LAM alone.

**Table:** Per participant analysis of test performance

**A) Compared to MGIT culture as a gold standard**					

	TB-LAMP *n/N* (%) [95% CI]	Xpert MTB/RIF *n/N* (%) [95% CI]	Smear microscopy *n/N* (%) [95% CI]	TB-LAM All CD4 counts *n/N* (%) [95% CI]	TB-LAM CD4 <200 cells/mm^3^ *n/N* (%) [95% CI]

Sensitivity	34/54 (63.0) [49.0–75.0]	40/54 (74.0) [60.4–84.3]	25/50 (50.0) [36.0–64.0]	7/53 (13.2) [6.3–25.7]	4/21 (19.0) [6.7–43.6]
Specificity	583/592 (98.5) [97.0–99.2]	577/592 (97.5) [95.8–98.5]	491/495 (99.2) [97.9–99.7]	494/503 (98.2) [96.6–99.1]	160/164 (97.6) [93.6–99.1]
PPV, % (95% CI)	79.1 (63.7–89.0) 72.7	(59.1–83.1)	86.2 (67.0–95.0)	43.8 (20.4–70.2)	50.0 (14.3–85.7)
NPV, % (95% CI)	96.7 (94.9–97.9) 97.6	(96.0–98.6)	95.2 (93.0–96.7)	91.5 (88.8–93.6)	90.4 (85.0–94.0)

**B) Compared to Xpert MTB/RIF as diagnostic standard**					

	TB-LAMP *n/N* (%) [95% CI]	Smear microscopy *n/N* (%) [95% CI]	TB-LAM all CD4 counts CD4 *n/N* (%) [95% CI]		TB-LAM <200 cells/mm^3^ *n/N* (%) [95% CI]

Sensitivity	37/55 (67.2) [53.5–78.6]	28/54 (51.9) [38.3-65.1]	9/50 (18.0) [9.4–31.7]		6/24 (25.0) [11.0–47.4]
Specificity	585/591 (99.0) [97.8–99.5]	490/491 (99.8) [98.6–100]	499/506 (98.6) [97.1–99.3]		159/161 (98.8) [95.1–99.7]
PPV, % (95% CI)	86.0 (71.5–93.8)	96.6 (77.0–99.6)	56.3 (29.8–79.6)		75.0 (27.6–96.0)
NPV, % (95% CI)	97.0 (95.3–98.1)	95.0 (92.7–96.6)	92.4 (89.8–94.4)		89.8 (84.4–93.5)

**C) NAAT tests vs. smear-negative, culture-positive and smear-positive, culture-positive**					

	TB-LAMP		Xpert		
	
	Smear-negative, culture-positive *n/N* (%) [95% CI]	Smear-positive, culture-positive *n/N* (%) [95% CI]	Smear-negative, culture-positive *n/N* (%) [95% CI]		Smear-positive, culture-positive *n/N* (%) [95% CI]

Sensitivity	12/25 (48.0) [31.8–71.8]	20/25 (80.0) [58.2–92.0]	14/25 (56.0) [35.3–74.8]		25/25 (100)
Specificity	488/495 (98.6) [97.1–99.3]	501/520 (96.3) [94.3–97.7]	480/495 (97.0) [95.0–98.2]		491/520 (94.4) [92.1–96.1]
PPV, % (95% CI)	63.2 (38.0–82.7)	51.3 (35.3–67.0)	48.3 (30.1–67.0)		46.2 (33.2–60.0)
NPV, % (95% CI)	97.4 (95.6–98.5)	99.0 (97.6–99.5)	97.8 (96.0–98.8)		100

MGIT =Mycobacterial Growth Indicator Tube; LAMP=loop-mediated isothermal amplification; CI=confidence interval; LAM=lipoarabinomannan; PPV=positive predictive value ; NPV = negative predictive value; NAAT = nucleic acid amplification test.

### All TB-positive

**Figure 3:**
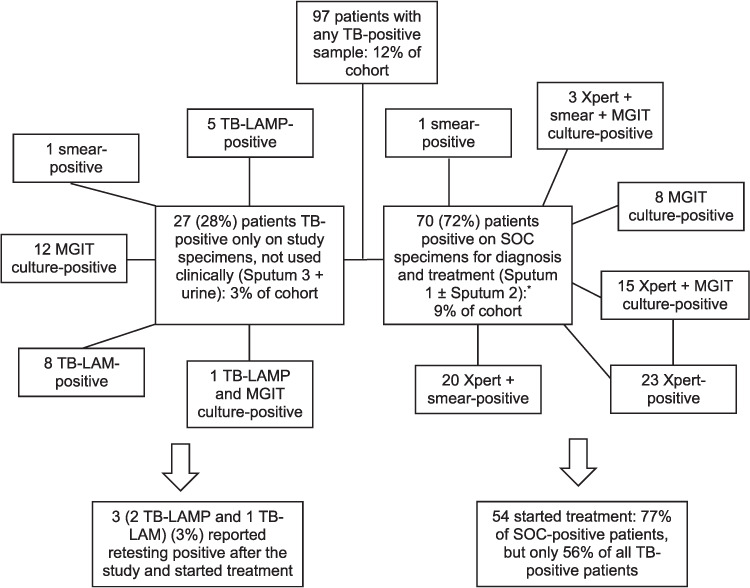
All HIV positive, ART-naïve patients with any positive TB test. ^*^20 were positive on SOC alone (14 Xpert, 5 SOC culture only and 1 smear only), 18 of which were negative on study samples and 2 did not have a study sample sent. LAMP = loop-mediated isothermal amplification; MGIT =Mycobacterial Growth Indicator Tube (liquid culture); SOC=standard of care; LAM=urine lipoarabinomannan; ART =antiretroviral therapy.

**Figure 4:**
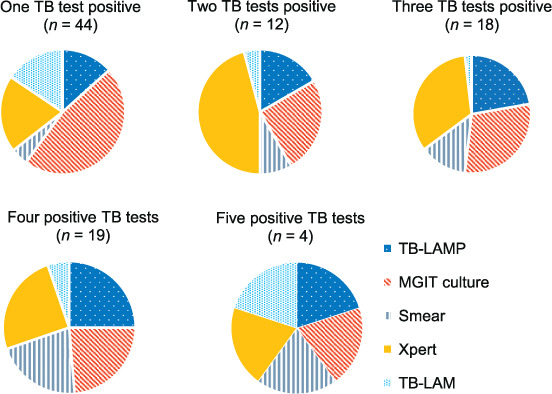
Positive TB test categories for the HIV-positive, ART-naive participants with any TB test positive (n = 97). All tests contribute to diagnosis, with culture predominating where one test is positive and Xpert where two or three tests are positive. LAMP = loop-mediated isothermal amplification; MGIT = Mycobacterial Growth Indicator Tube (liquid culture); LAM = urine lipoarabinomannan; ART =antiretroviral therapy.

Figure 3 shows all patients with any TB positive test performed (all tests are WHO-approved to initiate treatment in this population). This increased the number of TB-positives detected using SOC from 70/783 (9%) to a total of 97/783 (12%) for the cohort. The pooled culture positivity of all patients with sputum cultures was 64/95 (67%); 61/90 (71%) were detected using Xpert. In the 27 additional TB-positive patients who were detected only in study specimens, 14 had samples per DoH guidelines and the remaining had only study specimens. Figure 4 shows the distribution of positive tests.

### TB treatment

Of 97 patients, 70 (72%) were diagnosed using SOC tests, 57 (81%) of whom were known to have started TB treatment (three after retesting) (Figure 3). This constituted only 57/97 (59%) of all those with positive TB tests.

### Drug resistance testing

Of the 703 Xpert tests performed, 61/703 (7%) were positive, 4 were rifampicin-resistant, and 1 had an inconclusive result, which was subsequently confirmed to be not resistant using LPA. LPA was performed in 52/97 patients with positive specimens, which led to the identification of three additional specimens with isoniazid (INH) mono-resistance and one resistant to both rifampicin and INH that was Xpert-negative and culture-positive. Thus, 8/97(8%) had drug-resistant TB, 3 were treated for MDR-TB and 1 died before treatment initiation. Thus, only half of patients with resistant TB were detected using Xpert, and only half commenced treatment. Two of the 8 patients with drug-resistant TB reported prior TB treatment in the last 2 years.

### TB guidelines

For unknown reasons, DoH TB guidelines for PLHIV were not adhered to in 393/783 (50%) of patients. A second specimen for culture was not reported in 296/703 (38%) Xpert-negative patients and Xpert was not performed in 80/783 (10%). A second specimen for smear microscopy was not reported in 17/61 (28%) Xpert-positive patients. Missing samples that were repeated and processed after the initial date of enrolment were included in the analysis.

## DISCUSSION

This evaluation of TB-LAMP and TB-LAM against the SOC Xpert testing and culture was not conducted as per WHO validation standards, as TB-LAMP and Xpert testing were performed on different sputum samples. As sputum culture is a sub-optimal gold standard for TB disease (66% sensitivity compared to a clinical case definition),^14^ further consideration regarding optimal reference standards is required. TB is underdiagnosed in PLHIV as is clear from postmortem studies.^15^–^17^ As all the tests included in the study have similar specificities and are WHO-approved, we took a pragmatic approach and classified all positive TB tests as “possible TB”.

The study took place in real-life PHC settings with ART-naïve PLHIV in the city of Durban, an area hyper-endemic for TB (828/100 000 population)^18^ and HIV (44.4% antenatal prevalence).^19^ The poor algorithm adherence and many specimen challenges could be due to the high patient load in this setting. 16 Although sputum induction was offered, 137/783 (17%) patients were excluded from the complete analysis due to missing sputum specimens, 80/783 (10%) of which were not Xpert tested. The inability to produce sputum and other issues with sputum specimens (leakage, missing samples, insufficient samples) show the gaps that exist in the standard screening and diagnosis process for TB and resistant TB. As reported by Wilson et al., TB diagnosis is often “against all odds” in South Africa.^20^

The TB diagnostic algorithm for PLHIV was adhered to in 50% of the study participants, an improvement on algorithm adherence in the XTEND study (24%),^21^ which highlights the need for simpler algorithms and ongoing healthcare worker training and oversight.

The higher number of males among TB-positive patients (38%) was expected, but the 31% males overall suggests that a low proportion of males are accessing HIV care, which is worrying, as it is likely that men not receiving care are contributing to the ongoing community spread of both HIV and TB.

The 28% of possible TB cases identified through additional study testing indicates that in cases of HIV and TB, “the more you look, the more you find”. The 59% who started TB treatment in this cohort is comparable to the WHO 2020 TB fact sheet for South Africa;^1^ however, with SOC testing alone, a treatment start rate of 81% was documented in registers for this cohort.

Other studies have reported a similar performance for TB-LAMP,^22^–^25^ with lower sensitivity, but higher specificity and PPV than Xpert. Comparisons are limited by the lack of CD4 data available in other studies. The large heterogeneity of results across studies makes interpretation difficult, but this study found TB-LAMP had a higher sensitivity than smear microscopy.

As NAAT sensitivities are suboptimal in HIV patients, culture is required in cases with negative NAAT results. The strength of TB-LAMP is that, unlike the Xpert assay, the same specimen can be used for culture or LPA; Xpert requires a second specimen (often unavailable) for culture.

TB-LAMP is a rapid (60 min), benchtop test with manual steps and an instrument that can be used in a simple clinic laboratory and operated by an entry-level technician.^13^ It does not require information technology infrastructure or temperature-controlled environment. While TB-LAMP lacks the resistance testing provided by Xpert, Xpert missed 50% of the patients with drug resistance in this study. If TB-LAMP is followed by culture and drug susceptibility testing as required, it can be a useful NAAT POCT for TB diagnosis in resource-limited settings with a high HIV prevalence.

All study participants provided a urine specimen for TB-LAM testing, which suggests that urine is a preferred specimen to sputum and no biosafety precautions are required. As CD4 counts were unknown at enrolment, testing was not limited to only those with advanced HIV disease (CD4 <200 cells/mm^3^) as recommended. This meant that TB-LAM had a low yield (18/639) in the cohort. It should be noted that 8/97 (8.2%) of the TB-positives were detected using urine TB-LAM alone (which were possibly extrapulmonary disease) with a median CD4 count of 79 cells/mm^3^, making TB-LAM a valuable addition in this vulnerable population with more advanced HIV disease. TB-LAM performance could not be accurately determined, as a composite clinical reference standard would be required for this. However, as reported by Lawn et al.,^10^ TB-LAM increases the diagnostic yield among PLHIV with low CD4 counts, and is a useful additional test given that TB mortality is linked to CD4 count.^26^

In South Africa, the estimated notification rate for TB in 2019 was 58%.^1^ Pre-treatment loss to followup rates ranged from 20% to 25% in many sub-Saharan African and high-burden countries in 2016–2018.^27^–^29^ On-site POCT for TB would allow for ‘test and treat’ at a single visit and would be the best practice for TB care, mitigating many of the preanalytical errors experienced in this cohort. This could be augmented for PLHIV by POCT CD4 testing to identify vulnerable patients with low CD4 counts, who are likely to have a higher TB incidence and a greater mortality risk (18% with CD4 <100 cells/mm^3^ compared to 7% with CD4 >500 cells/mm^3^). An improved TB-LAM assay, FujiLam SILVAMP TB-LAM (FujiFilm, Tokyo, Japan), has been shown to have improved sensitivity in HIV-positive and -negative subjects, and is useful in the detection of extrapulmonary disease.^30^–^32^

Limitations of this study include lack of clinical follow-up of the patients other than the extraction of treatment data from clinic registers, and NHLS database and follow-up calls if patients had not started treatment. Also, different samples were used for the TB-LAMP and SOC Xpert tests, and there was poor adherence to the diagnostic algorithm with missed testing. This study was conducted before the rollout of Xpert Ultra in South Africa, which may have increased the sensitivity of Xpert testing, although Xpert specificity would have been decreased.^33^

In conclusion, TB-LAMP showed improved sensitivity compared to smear microscopy and better specificity than Xpert; TB-LAM is a useful add-in test. The cohort demonstrates the difficulty in diagnosing TB in PLHIV due to pre-analytical errors and poor testing algorithm adherence. Additional testing yielded a third more TB cases. Onsite NAAT POCT for TB would resolve many of problems currently being faced, and this new paradigm is urgently needed for streamlined universal health coverage.
